# Bayesian models as a unified approach to estimate relative risk (or prevalence ratio) in binary and polytomous outcomes

**DOI:** 10.1186/s12982-015-0030-y

**Published:** 2015-06-20

**Authors:** Vanessa Bielefeldt Leotti Torman, Suzi Alves Camey

**Affiliations:** Department of Statistics, Federal University of Rio Grande do Sul (UFRGS), Porto Alegre, RS Brazil; Post-Graduate Program in Epidemiology, Federal University of Rio Grande do Sul (UFRGS), Porto Alegre, RS Brazil

**Keywords:** Bayesian models, Relative risk, Prevalence ratio, Common outcomes, Dependent data, Polytomous outcomes

## Abstract

**Background:**

Disadvantages have already been pointed out on the use of odds ratio (OR) as a measure of association for designs such as cohort and cross sectional studies, for which relative risk (RR) or prevalence ratio (PR) are preferable. The model that directly estimates RR or PR and correctly specifies the distribution of the outcome as binomial is the log-binomial model, however, convergence problems occur very often. Robust Poisson regression also estimates these measures but it can produce probabilities greater than 1.

**Results:**

In this paper, the use of Bayesian approach to solve the problem of convergence of the log-binomial model is illustrated. Furthermore, the method is extended to incorporate dependent data, as in cluster clinical trials and studies with multilevel design, and also to analyse polytomous outcomes. Comparisons between methods are made by analysing four data sets.

**Conclusions:**

In all cases analysed, it was observed that Bayesian methods are capable of estimating the measures of interest, always within the correct parametric space of probabilities.

**Electronic supplementary material:**

The online version of this article (doi:10.1186/s12982-015-0030-y) contains supplementary material, which is available to authorized users.

## Background

Much has been discussed about disadvantages of the odds ratio (OR) as a measure of association in cross-sectional studies, cohort studies and clinical trials [1–8], and instead of it, relative risk (RR) or prevalence ratio (PR) according to the study design are proposed. Although some authors suggest that it is enough the outcome to be rare (<10 %) between unexposed [4, 5, 9] or rare in the general population [10, 11], Torman [12] showed that OR is only a good approximation of PR or RR and therefore can be interpreted as such, when the outcome is rare in the two strata of exposure.

In the case of binary outcomes and independent data, several alternatives to logistic regression have been proposed. One of them is the log-binomial model [13, 14], a generalized linear model with binomial response and log link function. Another proposal is the use of Poisson regression with robust variance [15–17]. Since robust Poisson regression assumes that the outcome has a Poisson distribution, probabilities larger than 1 can be estimated [16]. However, there may be convergence problems when fitting the log-binomial model [16, 18]. The use of the log-binomial model as a first choice of analysis was recommended by some authors [17, 19, 20] who compared this and other methods through simulation and observed that, when the log-binomial model converges, the resulting estimates of RR or PR have better properties. The Bayesian approach for the log-binomial model was proposed as a way to solve the convergence problems and the median point estimator and equal-tail credible interval (CI) were recommended [21]. Torman and Camey (unpublished manuscript) explored other Bayesian estimators and their final recommendation was to use the mode as a point estimator and the same credible interval.

For binary outcomes from dependent data, as in cluster randomized clinical trials or multilevel modelling, only frequentist proposals are known by the authors. Zou and Donner [22] and Yelland et al. [23] proposed Poisson and log-binomial regression models estimated with Generalized Estimating Equations (GEE), and compared them through simulation. They both verified that the GEE log-binomial model may have convergence problems. Yelland et al. [23] also found convergence problems with the GEE Poisson model, though less frequently than the GEE log-binomial. Zou and Donner [22] stressed that the proposed GEE models should be used only if the number of clusters is greater than 50. Moreover, the authors identified that the GEE Poisson model can estimate probabilities greater than 1. In the analysis of dependent data, in addition to GEE based models (also called marginal models), mixed models (also called conditional models) can be adopted. Yelland et al. [24] noted that there may be convergence problems when using the mixed-effects log-binomial model and that solutions for this problem are lacking.

On the estimation of PR or RR with polytomous outcomes, only papers with frequentist approach were found. Camey et al. [25] evaluated the performance of separated log-binomial and robust Poisson models, where several dichotomous outcomes are created and the desired model is fitted for each one. Comparisons with estimates from multinomial logistic regression were carried out by simulation and the conclusion was that the proposed approaches are more accurate and precise. The final recommendation was that fit of separate log-binomial models should be tried first and only resort to separate robust Poisson regressions if convergence problems occur. However, when considering separate dichotomous outcomes, the true multinomial nature of the response is ignored and there is no guarantee that the coefficients found will produce valid probabilities for the reference category. Another proposal is the log-multinomial model [26], which considers the correct distribution of the outcome, however, it may face convergence problems and estimate probabilities outside the correct interval. This last problem was not expected since the correct distribution of the outcome is adopted.

In this paper it is intended to exemplify the use of the Bayesian approach for the log-binomial model with independent data and extend that approach to support dependent data and multinomial outcomes. For this purpose four examples will be used.

## Methods

### Independent data and binary outcome

Data from a cohort of 65 patients admitted to a hospital in Porto Alegre for acute decompensated heart failure (ADHF) are used to illustrate the estimation of RR on binary outcomes with independent data. The outcome is death in three days after admission. The predictors considered were sodium (mEq/L), septum (mm) and pulmonary artery systolic pressure (PASP, mmHg), all continuous. In addition to estimating the RR of predictors, it is intended to get a formula to calculate a risk of death score that can be used in a practical way at admission. Since the estimated probabilities should naturally measure risk of death, it was decided to use the fitted model itself as a formula to calculate the risk score.

The corresponding log-binomial model is given by:

$$ {\theta}_i=P\left({Y}_i\Big|{X}_{1i},{X}_{2i},{X}_{3i}\right) = exp\left({\beta}_0+{\beta}_1{X}_{1i}+{\beta}_2{X}_{2i}+{\beta}_3{X}_{3i}\right)\kern1.25em i=1,\dots, 65 $$

where *Y*_*i*_ = 1 if the i-th individual died within the follow-up period and 0 otherwise, *X*_1*i*_ is his sodium level, *X*_2*i*_ his septum measure and *X*_3*i*_ his measure of PASP. A very important feature of the log-binomial model, which can cause convergence problems, is the fact that the coefficients of the model must be restricted to the condition that *θ*_*i*_ ≤ 1, ∀ *i*, i.e., only where the probabilities of each individual be between 0 and 1. In this model, *exp*(*β*_1_), *exp*(*β*_2_) and *exp*(*β*_3_) are the respective RRs of predictors (in the specific case of this data set, which is a cohort).

Two frequentist approaches were fitted to these data, robust Poisson regression and log-binomial model, both using the R 3.0.0 [27] function glm, with the sandwich package [28] for robust Poisson; and the Bayesian approach for the log-binomial model using Markov Chain Monte Carlo (MCMC) with the OpenBugs 3.2.2 program together with the R BRugs package [29, 30]. To compare the predictive power of the probabilities estimated by each model, ROC curves were obtained with the R Epi package [31]. The codes for this and other models used in this article can be found in the supporting web material (Additional file 1).

### Dependent data and binary outcome

To illustrate RR and PR estimation with the existence of dependency between observations, two sets of data were used. The first is from a cluster clinical trial, introduced by Kerry and Bland [32]. The aim was to verify the effect of an intervention on the practice of requiring radiology examinations, used by general practitioners in a certain hospital. For this, 34 doctors were divided equally in intervention and control groups, and for each patient referred for X-ray, it was evaluated if the requirement was in compliance with the guidelines.

In this context, the mixed-effects log-binomial model is given by:

$$ {\theta}_{ij}=P\left({Y}_{ij}=1\Big|{X}_{ij,ui}\right) = exp\left({\beta}_0+{\beta}_1{X}_{ij}+{u}_i\right)\kern1.5em i=1,\dots, 34\kern1em j=1,\dots, {n}_i $$

where *Y*_*i j*_ = 1 if the j-th examination of the i-th doctor was in accordance with the guidelines and 0 otherwise, *X*_*i j*_ = 1 if the i-th the doctor was in the intervention group and 0 if it was in the control group, *u*_*i*_ is the effect due to the i-th doctor, for which it is supposed that *u*_*i*_ ~ *N*(0, *σ*_*u*_^2^) and *n*_*i*_ is the number of patients seen by the i-th physician. The GEE log-binomial model equation is the same equation of the independent data case, just no longer assuming independence between observations. In the GEE Poisson model, assumption of Poisson distribution is added to the outcome.

Three frequentist models were fitted to this data set, using SAS version 9.3 (SAS Institute, Cary NC): mixed-effects log-binomial model with Proc GLIMMIX, GEE log-binomial model and GEE Poisson model with Proc GENMOD using an exchangeable working correlation matrix. This matrix was chosen to make possible the estimation of the intraclass correlation coefficient (ICC) [33], which measures the degree of data dependence. The Bayesian approach for the mixed-effects log-binomial model was performed again with BRugs.

The second data set comes from a cross-sectional study with multilevel design on evaluation of the Unified Health System (Sistema Único de Saúde, SUS) [34] by the users. Data were collected by the SUS General Ombudsman Office Department (Departamento de Ouvidoria Geral do SUS) of the Strategic and Participatory Management Secretariat (Secretaria de Gestão Estratégica e Participativa) of the Ministry of Health (Ministério da Saúde), through telephone contact. The inclusion criteria were to be 16 years old or older and to have used SUS in the last 12 months. Respondents were inhabitants of 61 municipalities, and multilevel analysis was adopted to consider the expected dependence among individuals residing in the same municipality. The outcome was user dissatisfaction. Predictor variables related to municipalities and individuals were considered. For comparison of the different fits it was used the final model presented by the authors, obtained from a sample of 12,879 interviews. The mixed effects logistic regression was fitted using SAS Proc GLIMMIX and the mixed-effects log-binomial model estimated via MCMC. Here, the use of logistic regression aims to compare differences between OR and PR in a large sample. The equation of the mixed-effects log-binomial model is similar to the one in the cluster clinical trial example, adding predictors.

The extension of the log-binomial model to incorporate mixed effects through the Bayesian approach was made by adding a normally distributed random effect in the linear predictor of the model, which can be seen in the given code. This same term was added in the place where the restriction for probabilities between 0 and 1 is implemented in the MCMC code.

For the mixed-effects logistic model there is more than one way to estimate ICC, here we use the following formula [35]:

$$ IC{C}_{logist}=\frac{\sigma_u^2}{\sigma_u^2+\frac{\pi^2}{3}} $$

This expression is obtained by considering that the binary outcome comes from a continuous latent variable and that this one conforms to a model with residuals following the standard logistic distribution. Using the same reasoning for the mixed effects log-binomial model [see Additional file 1], a model is reached for the continuous latent variable with residuals following an exponential distribution with mean equals to 1, which leads to following formula for the ICC:

$$ IC{C}_{log- bin}=\frac{\sigma_u^2}{\sigma_u^2+1}, $$

easily estimated by point and interval estimators in the Bayesian approach. However, in the frequentist approach to logistic regression no direct way was found to obtain the confidence interval (CI) for the ICC in SAS. Likewise, in the trial data it was not found a way to estimate between clusters variance with GEE, nor to estimate the confidence interval of the ICC for this method.

### Independent data and polytomous outcome

The last database evaluated is one provided in the book of Hosmer and Lemeshow [36] on the birth weight of 189 live births. For illustration of the models with polytomous outcome, birth weight was divided into 4 categories (<2.5 kg, 2.5 kg to 3 kg, 3 kg to 4 kg, 4 kg or more) and the third one was considered the reference category. Thus, a binary variable can be defined for each category of the outcome: *Y*_*i j*_ = 1 if the i-th born had birth weight belonging to the j-th category, *j* = 0,1, …, 3, *j* = 0 being the reference category and *i* = 1, …, 189. Mother’s age (*X*_1*i*_) and her smoking condition during pregnancy (*X*_2*i*_) were used as predictors.

The log-multinomial model defined in this context is:

$$ {\theta}_{ij}=P\left({Y}_{ij}=1\Big|{X}_{1i},{X}_{2i}\right)= \exp \left({\beta}_{j0}+{\beta}_{j1}{X}_{1i}+{\beta}_{j2}{X}_{2i}\right)\kern0.75em j=1,\dots, 3. $$

It is redundant to estimate coefficients for the reference category (*j* = 0) since

$$ {Y}_{i0}=1-{\displaystyle {\sum}_{j=1}^3{Y}_{ij}},\ \forall i,\ \mathrm{then}\ P\left({Y}_{i0}=1\Big|{X}_{1i},{X}_{2i}\right)=1-{\displaystyle {\sum}_{j=1}^3P\left({Y}_{ij}=1\Big|{X}_{1i},{X}_{2i}\right)},\ \forall i $$

Three frequentist analyzes were performed on these data: separated robust Poisson regressions, separate log-binomial models and log-multinomial model. The log-multinomial model was fitted in Stata version 9.2 (Stata Corp, College Station, Texas) with syntax courtesy of Leigh Blizzard. Separate regressions were performed in R, the same way as in the case of binary outcomes. To implement the log multinomial model via MCMC, in addition to the restriction for probabilities to be between 0 and 1, it is necessary to restrict the probability of the reference category be the complement of the sum of the probabilities of the other categories [see Additional file 1].

### Details common to all the examples

To choose the numbers of interactions, burn-in period and thin for MCMC, graphical analysis and Gelman and Rubin statistic [37] were used. At least 1000 iterations were used for estimation. The prior distributions assigned to the models coefficients were normal with zero mean and variance 10^6^ as suggested by Chu and Cole [21]. In the case of models for dependent data, the uniform distribution from 0.01 to 100 for the deviation of the random effects was adopted, as suggested by Gelman [38] in the context of normal mixed models. The priors used are all vague in order to minimize their influence on the results. The mode and the equal tails credible interval were used as Bayesian point and interval estimators, respectively.

For all analyzes, point and 95 % confidence/credible interval estimates will be shown. For comparisons between methods, the ranges of the intervals were calculated. For comparison of point estimates, one of the frequentist methods was adopted as a reference and the relative difference in percentage (Δ %) from the other methods was calculated. Additional information about computational time will also be presented for the Bayesian models considering running in a computer with 3.40 GHz processor and 4 GB RAM.

## Results

For the Bayesian models, all the chains simulated were considered well mixed after the chosen burn-in and thin was applied. This was checked through comparison of the trajectory plots before and after the discard of some generated values. Also, the Gelman and Rubin statistics was near to one in all situations. Details of convergence check are shown for the cluster randomized trial in the supporting web material (Additional file 1) as an example.

### Independent data and binary outcome

In the cohort of 65 patients with heart failure, 15 died (23.1 %), therefore, OR may not be a good approximation to RR. Table 1 shows the results of the analyses performed. The frequentist log-binomial model did not converge and therefore does not appear. It can be observed that the point estimates of the coefficients differed more than the estimates of RRs, with percentage differences ranging from 12.5 to 38.6 %. Except for the intercept, the ranges of the intervals differed in the second decimal place, and the robust Poisson regression generally had a shorter range. In terms of significance of predictors, the methods diverged only on PASP, which was considered significant by robust Poisson regression and not significant by the Bayesian method.

**Table 1:** Results of the ADHF patients cohort analyses

Parameter	Point and 95 % CI by Method		∆ % ^2^	Range of CI by Method	
	Robust Poisson	MCMC^1^		Robust Poisson	MCMC
Intercept	9.058 (1.124; 16.992)	7.742 (−2.670; 13.980)	−14.526	15.868	16.650
Septum Coefficient	0.229 (0.011; 0.446)	0.184 (0.017; 0.431)	−19.580	0.435	0.414
Sodium Coefficient	−0.100 (−0.159; −0.042)	−0.088 (−0.136; −0.009)	12.521	0.116	0.126
PASP Coefficient	0.018 (0.001; 0.036)	0.011 (−0.015; 0.027)	−38.650	0.035	0.042
Septum RR	1.257 (1.012; 1.562)	1.196 (1.018; 1.539)	−4.860	0.551	0.521
Sodium RR	0.904 (0.853; 0.958)	0.915 (0.873; 0.991)	1.135	0.105	0.118
PASP RR	1.018 (1.001; 1.036)	1.011 (0.986; 1.028)	−0.711	0.036	0.042

^1^Random effects log-binomial model, mode point estimator and equal tails interval. CPU time: 24s. Details of MCMC simulation: 3 chains, 50000 iterations in each one plus the first 50000 that were discarded, and a thin of 100 iterations was applied

^2^ $$ \varDelta \%=\left(\frac{\mathrm{MCMC}\ \mathrm{point}\ \mathrm{estimate} - \mathrm{Poisson}\ \mathrm{point}\ \mathrm{estimate}}{\left|\mathrm{Poisson}\ \mathrm{point}\ \mathrm{estimate}\right|}\right) $$

**Fig. 1:**
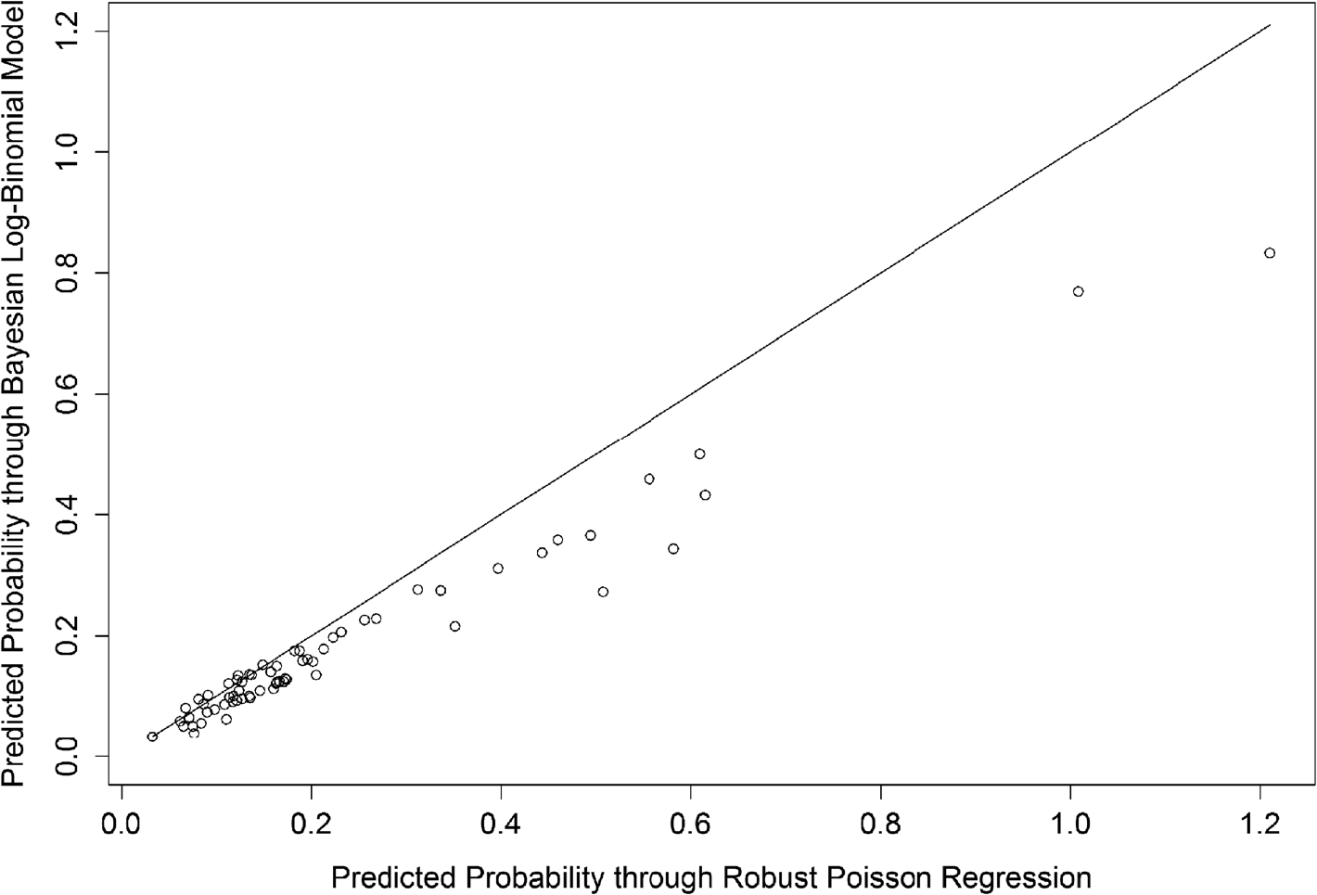
Scatterplot of probabilities predicted through robust Poisson regression versus MCMC Log-binomial and straight line of equality

**Fig. 2:**
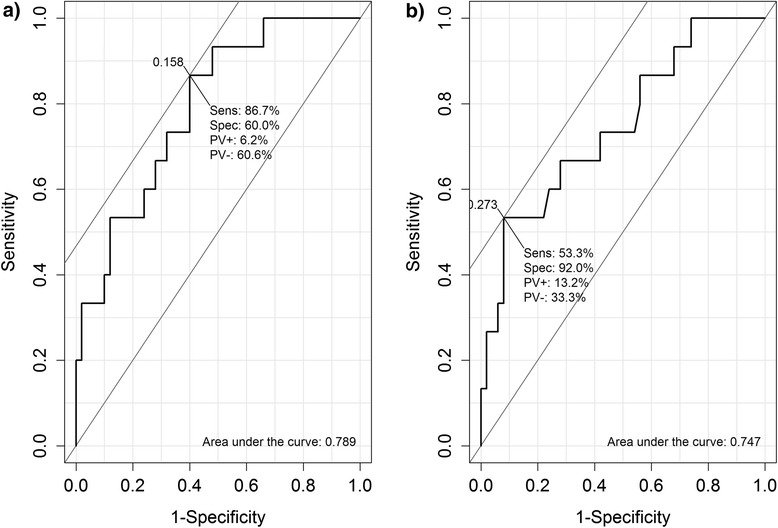
ROC curve of probabilities predicted through (**a**) Robust Poisson regression and (**b**) Bayesian log-binomial model

Figure 1 shows a scatter plot comparing the probabilities predicted by each method. Although there is a high correlation (r = 0.984), it is apparent that the predicted probabilities are different since there are several points distant from the straight line of equality. It is also apparent that robust Poisson produced two unacceptable estimates of probability, i.e., greater than 1. An individual with 13 mm septum, 136 mEq/L of sodium and 100 mmHg of PASP got a 1.211 probability of death predicted by Poisson and another individual with 12 mm septum, 127 mEq/l of sodium and 53 mmHg of PASP got a probability of death estimated in 1.008. All those values are outside the normality ranges recommended, but are plausible. Both patients died. Figure 2 shows the ROC curves of the probabilities estimated by Poisson regression and by the log-binomial model via MCMC. Probabilities predicted by Poisson regression have an area under the curve slightly larger than the Bayesian method, but the optimal cutoff point of the Bayesian method is higher.

### Dependent data and binary outcome

In the cluster clinical trial presented in Kerry and Bland [33], among the 429 requests in the intervention group, 341 (79.48 %) were in compliance with the guidelines. Now, among the 702 requests in the control group, 509 (72.51 %) were adequate. In Table 2 the results of the models fitted for these data are shown. The results of the GEE Poisson model were virtually identical to the GEE log-binomial and so were deleted. Convergence was not obtained when fitting the mixed-effects log-binomial model via SAS. The largest difference was observed in the point estimate of the ICC. The ranges of CIs were slightly wider in the Bayesian approach. Both methods detected significant effect of the intervention.

**Table 2:** Results of the analyses of the cluster clinical trial on guidelines for radiology requests

Parameter	Point and 95 % CI by Method		∆ % ^2^	Range of CI by Method	
	Log-binomial GEE	MCMC^1^		Log-binomial GEE	MCMC
Intercept	−0.315 (−0.371; −0.259)	−0.314 (−0.387; −0.256)	0.412	0.112	0.131
Intervention Coefficient	0.092 (0.007; 0.178)	0.089 (0.006; 0.183)	−3.684	0.171	0.177
Intervention RR	1.097 (1.007; 1.195)	1.089 (1.006; 1.201)	−0.693	0.188	0.195
Random effect variance	-	0.007 (0.001; 0.020)	-	-	0.019
ICC	0.010	0.007 (0.001; 0.020)	−26.042	-	0.019

^1^Random effects log-binomial model, mode point estimator and equal tails interval. CPU time: 100s. Details of MCMC simulation: 3 chains, 210000 iterations in each one plus the first 50000 that were discarded, and a thin of 600 iterations was applied

^2^ $$ \varDelta \%=\left(\frac{\mathrm{MCMC}\ \mathrm{point}\ \mathrm{estimate}\kern0.5em  - \mathrm{G}\mathrm{E}\mathrm{E}\ \mathrm{Log}-\mathrm{binomial}\ \mathrm{point}\ \mathrm{estimate}\ }{\left|\mathrm{G}\mathrm{E}\mathrm{E}\ \mathrm{Log}-\mathrm{binomial}\ \mathrm{point}\ \mathrm{estimate}\right|}\right) $$

Table 3 presents estimates based on the SUS users’ satisfaction survey data. Among the respondents, 7,875 (61.15 %) were classified as dissatisfied with the SUS. Large differences can be noted between logistic regression’s OR and the PR estimated by log-binomial model via MCMC. Range of Bayesian CI was shorter for all parameters. The methods differed only on the significance of three variables: percentage of literate population, health units per hundred thousand inhabitants and graduate or higher education level and the CI obtained by MCMC for all of them included the value 1 while that obtained by logistic regression excluded it.

**Table 3:** Results of multilevel analysis of the SUS users’ satisfaction data

Parameter	Point and 95 % CI by Method		∆ %^3^	Range of CI by Method	
	Logistic^1^	MCMC^2^		Logistic	MCMC
Population density (km^2^/thousand inhab.) PR	1.026 (0.992; 1.061)	1.008 (0.999; 1.017)	−1.798	0.069	0.018
% Literate population PR	1.061 (1.017; 1.106)	1.010 (0.998; 1.021)	−4.834	0.089	0.023
Per capta income (thousands of reais) PR	0.859 (0.760; 0.971)	0.963 (0.927; 0.999)	12.163	0.211	0.072
Poverty PR	1.006 (0.998; 1.014)	1.001 (0.999; 1.003)	−0.479	0.016	0.004
Human development index PR	0.027 (0; 1.922)	0.514 (0.188; 2.035)	1803.236	1.922	1.847
Health Units per one hundred thousand inhab. PR	0.981 (0.965; 0.998)	0.995 (0.991; 1.000)	1.426	0.033	0.009
Coverage of the Family Health Strategy PR	1.006 (1.002; 1.009)	1.001 (1.000; 1.003)	−0.452	0.007	0.003
SUS Index PR	0.940 (0.819; 1.078)	0.982 (0.945; 1.024)	4.507	0.259	0.079
Age PR					
Up to 20 years	0.968 (0.811; 1.157)	0.969 (0.911; 1.047)	0.133	0.346	0.136
21 to 30 years	1.320 (1.135; 1.535)	1.068 (1.022; 1.137)	−19.056	0.400	0.115
31 to 40 years	1.277 (1.066; 1.483)	1.062 (1.016; 1.127)	−16.872	0.384	0.111
41 to 50 years	1.184 (1.013; 1.384)	1.048 (1.004; 1.116)	−11.522	0.371	0.112
51 to 60 years	1.133 (0.958; 1.341)	1.046 (0.996; 1.113)	−7.706	0.383	0.117
More than 60 years	-	-	-	-	-
White color PR	1.084 (0.998; 1.177)	1.011 (0.990; 1.028)	−6.701	0.179	0.038
Education PR					
Illiterate	-	-	-	-	-
Literate	0.964 (0.686; 1.357)	0.999 (0.892; 1.125)	3.626	0.671	0.233
Elementary	1.150 (0.818; 1.617)	1.063 (0.951; 1.187)	−7.576	0.799	0.236
High	1.293 (0.921; 1.815)	1.089 (0.966; 1.204)	−15.808	0.894	0.238
Higher	1.665 (1.163; 2.385)	1.098 (0.987; 1.238)	−34.059	1.222	0.251
Not attended at home PR	1.488 (1.372; 1.613)	1.092 (1.064; 1.122)	−26.618	0.241	0.058
End of attendance PR					
Resolved	-	-	-	-	-
Partially Resolved	1.957 (1.785; 2.146)	1.282 (1.245; 1.324)	−34.475	0.361	0.079
Not Resolved	3.726 (3.260; 4.257)	1.366 (1.318; 1.409)	−63.348	0.997	0.091
Time for attendance PR					
Up to 30 min.	-	-	-	-	-
Up to 1h	1.304 (1.179; 1.443)	1.115 (1.075; 1.162)	−14.527	0.264	0.087
Up to 4h	1.782 (1.626; 1.952)	1.205 (1.172; 1.255)	−32.356	0.326	0.083
More than 4h	2.547 (2.157; 3.008)	1.233 (1.188; 1.280)	−51.586	0.851	0.092
Random effect variance	0.052 (0.031; 0.106)	0.003 (0.002; 0.007)	−93.255	0.075	0.005
ICC	0.015	0.003 (0.002; 0.007)	−77.531	-	0.005

^1^Random-effects logistic model, OR estimates. ^2^ Random effects log-binomial model, mode point estimator and equal tails interval. Approximate CPU time 1 week. Details of MCMC simulation: 3 chains, 480000 iterations in each one plus the first 250000 that were discarded, and a thin of 400 iterations was applied.^3^ $\varDelta \%=\left(\frac{\mathrm{MCMC}\ \mathrm{point}\ \mathrm{estimate}\ \hbox{--} \kern0.5em \mathrm{Logistic}\ \mathrm{point}\ \mathrm{estimate}}{\left|\mathrm{Logistic}\ \mathrm{point}\ \mathrm{estimate}\right|}\right)$

### Independent data and polytomous outcome

Among 189 births in the database taken from Hosmer and Lemeshow [36], 59 (31.22 %) weighed below 2.5 kg, 38 (20.10 %) between 2.5 and 3 kg, 83 (43.92 %) between 3 and 4 kg, and 9 (4.76 %) of 4 kg or more.

The results of the analyses conducted are shown in Table 4 and comparative measurements in Table 5. When fitting the log-binomial model for category 4 kg or more there was a non-convergence message, but results were produced and it was decided to show them. In general, the separated log-binomial models produced the more similar to the log-multinomial model estimates. As for the directions of associations, no discrepancy occurred. However, for the last category of the outcome, the Bayesian method was the only one which identified the association of smoking as significant and the only one which did not identify association of age as significant. Bayesian and log-multinomial methods produced, in general, wider range intervals.

**Table 4:** Results of the analyses of the low birth weight data with multinomial outcome

Parameter	Point and 95 % CI for each Method			
	Separate Poisson	Separate Log-binomial	Log-multinomial	MCMC^1^
*Outcome Weight up to 2.5 kg*				
Intercept	−0.596 (−1.554; 0.363)	−0.683 (−1.688; 0.322)	−0.667 (−1.673; 0.340)	−0.813 (−1.748; 0.278)
Smoke Coefficient	0.461 (0.042; 0.879)	0.444 (0.027; 0.861)	0.439 (0.021; 0.857)	0.430 (0.009; 0.872)
Age Coefficient	−0.034 (−0.074; 0.006)	−0.030 (−0.073; 0.013)	−0.031 (−0.073; 0.012)	−0.025 (−0.073; 0.012)
Smoke RR	1.585 (1.043; 2.410)	1.559 (1.028; 2.365)	1.551 (1.021; 2.355)	1.459 (1.009; 2.392)
Age RR	0.966 (0.929; 1.006)	0.971 (0.930; 1.013)	0.970 (0.930; 1.012)	0.975 (0.930; 1.012)
*Outcome Weight from 2.5 to 3 kg*				
Intercept	−2.247 (−3.573; −0.920)	−2.244 (−3.536; −0.953)	−2.288 (−3.619; −0.957)	−2.486 (−3.578; −1.045)
Smoke Coefficient	0.136 (−0.438; 0.710)	0.138 (−0.435; 0.711)	0.154 (−0.419; 0.728)	0.196 (−0.470; 0.732)
Age Coefficient	0.025 (−0.027; 0.077)	0.025 (−0.026; 0.075)	0.026 (−0.026; 0.078)	0.032 (−0.024; 0.070)
Smoke RR	1.146 (0.645; 2.034)	1.147 (0.647; 2.035)	1.167 (0.657; 2.071)	1.005 (0.625; 2.078)
Age RR	1.025 (0.973; 1.080)	1.025 (0.975; 1.078)	1.027 (0.975; 1.081)	1.032 (0.976; 1.073)
*Outcome Weight above 4 kg*				
Intercept	−5.474 (−7.629; −3.319)	−6.122 (−7.758; −4.485)	−6.079 (−9.217; −2.940)	−4.890 (−7.082; −2.616)
Smoke Coefficient	−1.545 (−3.557; 0.488)	−1.572 (−3.635; 0.490)	−1.478 (−3.517; 0.560)	−1.536 (−5.006; −0.122)
Age Coefficient	0.111 (0.041; 0.181)	0.136 (0.099; 0.173)	0.133 (0.025; 0.241)	0.097 (−0.003; 0.150)
Smoke RR	0.216 (0.029; 1.629)	0.208 (0.026; 1.633)	0.228 (0.030; 1.751)	0.054 (0.006; 0.886)
Age RR	1.117 (1.042; 1.198)	1.146 (1.105; 1.188)	1.142 (1.025; 1.272)	1.102 (0.997; 1.162)

^1^Log-multinomial model, mode point estimator and equal tails interval. CPU time 52 h. Details of MCMC simulation: 3 chains, 3012000 iterations in each one plus the first 30000 that were discarded, and a thin of 3000 iterations was applied

**Table 5:** Comparisons among analyses of the low birth weight data with multinomial outcome

Parameter	∆ %^1^/ Range of 95 % CI by Method			
	Separate Poisson	Separate Log-binomial	Log-multinomial	MCMC
*Outcome Weight up to 2.5 kg*				
Intercept	10.675 / 1.917	−2.455 / 2.009	- / 2.013	−21.967 / 2.026
Smoke Coefficient	5.008 / 0.837	1.207 / 0.833	- / 0.836	−1.975 / 0.863
Age Coefficient	−11.694 / 0.079	1.988 / 0.086	- / 0.084	19.127 / 0.085
Smoke RR	2.222 / 1.367	0.531 / 1.337	- / 1.334	−5.927 / 1.383
Age RR	−0.356 / 0.077	0.061 / 0.083	- / 0.082	0.505 / 0.082
*Outcome Weight from 2.5 to 3 kg*				
Intercept	1.810 / 2.653	1.907 / 2.582	- / 2.663	−8.665 / 2.533
Smoke Coefficient	−11.918 / 1.148	−10.876 / 1.146	- / 1.148	26.956 / 1.202
Age Coefficient	−5.462 / 0.104	−5.918 / 0.101	- / 0.104	19.877 / 0.095
Smoke RR	−1.822 / 1.388	−1.664 / 1.388	- / 1.414	−13.865 / 1.453
Age RR	−0.144 / 0.106	−0.156 / 0.104	- / 0.106	0.473 / 0.097
*Outcome Weight above 4 kg*				
Intercept	9.942 / 4.310	−0.707 / 3.274	- / 6.278	19.558 / 4.466
Smoke Coefficient	−3.794 / 4.045	−6.350 / 4.125	- / 4.077	−3.888 / 4.944
Age Coefficient	−16.773 / 0.140	2.304 / 0.073	- / 0.216	−26.748 / 0.153
Smoke RR	−5.454 / 1.600	−8.961 / 1.606	- / 1.721	−76.265 / 0.879
Age RR	−2.205 / 0.156	0.307 / 0.084	- / 0.247	−3.501 / 0.165

^1^ $$ \varDelta \%=\left(\frac{\mathrm{point}\ \mathrm{estimate}\ \mathrm{of}\ \mathrm{the}\ \mathrm{method}\ \hbox{--}\ \mathrm{Log}-\mathrm{multinomial}\ \mathrm{point}\ \mathrm{estimate}\ }{\left|\mathrm{Log}-\mathrm{multinomial}\ \mathrm{point}\ \mathrm{estimate}\right|}\right) $$

Making up predictions of probabilities for each Poisson regression, no case of probability greater than 1 occurred. However, when adding the predicted probabilities for the three outcomes, in one case a value greater than 1 is obtained. The same occurred for the separate log-binomial models and for the log-multinomial model.

## Discussion

Some interesting features learned from the analysis performed are worth mentioning. For the cohort of patients with ADHF, a low degree of agreement between the probabilities estimates by the Bayesian and the frequentist method was observed. This was expected since the largest differences were found in the point estimation of the coefficients. The MCMC log-binomial model produced lower probabilities estimates. This shrinkage happens probably because of the correct parametric restriction. Poisson regression can estimate probabilities greater than one so the probabilities are inflated for this data. Also, the Bayesian method produced a higher optimal cutoff point in the ROC curve, which was more coherent with the outcome (death).

For the SUS example, the random effects logistic regression can produce estimates of OR quite distant from the PR, and all of its estimates overestimate the association of predictors, a property already widely discussed about OR in the context of independent data [2, 4–9, 11].

For the birth data from Hosmer and Lemeshow [36], regardless of the reference category, at least two other outcomes cannot be considered rare (occurred for more than 10 % of the sample). This fact is very likely to occur with at least one of the categories in polytomous data. So, for polytomous data, there will be very few situations in each the OR will be a good approximation of RR or PR.

Also for this dataset, it was observed that all the frequentist methods estimated probabilities greater than one for the reference category. So, only the Bayesian model can be used to obtain valid predicted probabilities for the reference outcome.

It is important to stress that the choice of the reference category for the outcome in the log-multinomial model will not affect the interpretation of the RRs of the other categories, unlike what happens in the multinomial logistic regression. The chosen category must be the one for which identifying associated predictors is of no interest. However, in the separate models it is possible to estimate also a model for that reference category, but this is not interesting because it does not enable comparisons with the log multinomial model.

## Conclusions

The Bayesian approach was presented in this paper as a unified way to estimate relative risk (or prevalence ratio) for situations with binary outcomes and dependent or independent data and for polytomous outcomes in independent data. It was not illustrated here, but the extension to the case of polytomous outcomes and dependent data can be made with the same fit made for dichotomous outcomes.

It was shown that the Bayesian approach overcomes difficulties of convergence common in the frequentist approach for the log-binomial model. It correctly restricts the parameter space to produce valid probabilities, which is especially fundamental in cases such as the study of patients with ADHF where, besides associated predictors being known, it is desired to build a prediction score. Chu and Cole [21] showed that in addition, a restriction for a space of values of covariates not observed in the sample can be programmed, so even for values of the predictors of patients outside the study, the estimated probabilities may be valid.

For dependent data, the proposed Bayesian method overcomes the difficulty of convergence of the mixed-effects log-binomial model. Besides, it has the advantage that it allows obtaining estimates of the random effect variance (point and interval) and the ICC interval more directly than the GEE method. An expression to calculate the ICC for random effects log-binomial models was also proposed. Such expression still needs to be compared with the ones proposed by Yelland et al. [24].

For polytomous data, it was seen that the separate methods and the log-multinomial model may not produce valid probabilities for the reference category of the outcome. Even though it did not occur in this work, the use of separate Poisson regressions and the log-multinomial model can still result in invalid probabilities for the outcomes analyzed. The log-multinomial model may still face convergence problems, and is only implemented in a commercial computer application (Stata). The proposed MCMC methodology produced coherent estimates and through the use of free programs (R and OpenBugs).

A limitation of the Bayesian approach is that the computational time can be quite large. This occurred especially with the data on low birth weight and on satisfaction about SUS. In the first one there was a high correlation between the values generated, a large number of values had to be generated to get a reasonable sample for estimation, even using a high thin. In the second one, the huge sample size and the large number of parameters to be estimated were responsible for a slow performance of the routine. One alternative tested to overcome this problem was the use of Laplace integration [39] by means of the package INLA [40], which has the advantage of being much faster than MCMC. The method worked very well for the examples with dependent data, but for the patients with ADHF data it produced probabilities greater than 1, and therefore the results were not shown. More studies are necessary to understand whether this limitation can be overcome. Another alternative that can be investigated is the use of other MCMC softwares, like JAGS [41] and the more recent STAN [42]. The modification of the considered priors can also be an alternative to improve the performance of the Bayesian approach, especially relating the variance of the random effect. Regarding the priors of the coefficients, Chu and Cole [21] also evaluated the normal priori with variance 10^2^ and concluded that it produced results very similar to that of variance equal to 10^6^.

A limitation of this study is that only empirical comparisons between methods were proceeded. So, strong recommendations about which method is usually better cannot be made. Bayesian methods appeared to be promising since they can deal correctly with the probabilities involved in analyzing both binary and polytomous outcomes. Simulations were performed by Chu and Cole [21] and Torman and Camey (unpublished manuscript) only for the case of binary outcome and independent data. Plan is to make simulation studies also for situations with binary outcome and dependent data and for polytomous outcomes and independent and dependent data.

## Additional file

Additional file 1:
**Supporting Web Material.** PDF document with R codes for analyses and with the demonstration of the ICC’s formula.

## Abbreviations

OR: Odds Ratio
RR: Relative Risk
PR: Prevalence Ratio
CI: Credible/confidence interval
GEE: Generalized Estimating Equations
ADHF: Acute Decompensated Heart Failure
PASP: Pulmonary Artery Systolic Pressure
MCMC: Markov Chain Monte Carlo
ICC: Intraclass Correlation Coefficient
SUS: Sistema Único de Saúde
