# Supplementary material for: Bayesian models as a unified approach to estimate relative risk (or prevalence ratio) in binary and polytomous outcomes
Source: Emerg Themes Epidemiol. 2015 Jun 20;12:8. doi: 10.1186/s12982-015-0030-y (PMC4473845; doi:10.1186/s12982-015-0030-y)
Supplement: Additional file 1: — Supporting Web Material. PDF document with R codes for analyses and with the demonstration of the ICC’s formula. [file 12982_2015_30_MOESM1_ESM.pdf]

SUPPORTING WEB MATERIAL

1 Code for the ADHF patients data

```
###################### BAYESIAN ANALYSIS IN R ######################

# Importing SPSS dataset
library(foreign)
ADHF = read.spss("banco_ICAD.sav", to.data.frame=TRUE)

# Excluding missings
attach(ADHF)
ADHFcompl = na.omit(data.frame(óbito, Septo.mm, Na.baseline, PSAP))

# Loading BRugs package
library(BRugs)

# Defining dataset for BRugs
ADHFbugs = list(N=65, Y=ADHFcompl$óbito , X1 = ADHFcompl$Septo.mm, X2
= ADHFcompl$Na.baseline, X3=ADHFcompl$PSAP)

# Defining log-binomial model for BRugs
model.log.bin = function(){
for(i in 1:N){
      Y[i] ~ dbern(p[i])
      log(p[i]) <- a0 + a1*X1[i]+ a2*X2[i]+ a3*X3[i]
}
    # Priors
a0 ~dnorm(0,1.0E-6)
a1 ~dnorm(0,1.0E-6)
a2 ~dnorm(0,1.0E-6)
a3 ~dnorm(0,1.0E-6)
    # Calculation of RRs
    RR1 <-exp(a1)
    RR2 <-exp(a2)
    RR3 <-exp(a3)
    # Implementing restriction for probabilities between 0 and 1.
for (i in 1:N){
      ones[i] <- 1
      ones[i]  ~ dbern(C1[i])
      C1[i] <- step(1-p[i])
}
}

# Identifying parameters for which estimates are wanted
parameters<- c("a0","a1","a2","a3","RR1","RR2","RR3","p")

# Setting initial values for 3 chains, they must produce valid
probabilities, negative numbers are recommended for the intercept
inits<- list(
      list(a0 =-10, a1=0, a2=0, a3=0),
      list(a0 =-20, a1=0, a2=0, a3=0),
      list(a0 =-30, a1=0, a2=0, a3=0)
)

# Running MCMC simulations
simul<- BRugsFit(data=ADHFbugs,  para=parameters, inits=inits,
      modelFile=model.log.bin, numChains=3, nIter=50000, nThin=100,
      nBurnin=50000)

#################### FREQUENTIST ANALYSIS IN R ####################
```

```
########## Robust Poisson

# Loading packages lmtest and sandwich
library(lmtest)
library(sandwich)

# Fitting the model and getting robust variance estimates
modelpr <- glm(óbito ~ Septo.mm + Na.baseline + PSAP ,family=poisson,
data=ADHFcompl)
coefs = coeftest(modelpr,vcov=sandwich)

########## Log-binomial

# Attempt to fit the model (convergence not achieved)
modellog<- glm(óbito ~ Septo.mm + Na.baseline + PSAP,
family=binomial(link=log), data=ADHFcompl)
```

2 Code for the cluster clinical trial data with convergence check and estimation of mode for the Bayesian model

```
###################### BAYESIAN ANALYSIS IN R ######################

# Defining dataset for BRugs, as shown in Kerry and Bland (with number
of successes and total number of patients per physician)

NTrials = c(20,
          7,
          16,
          31,
          20,
          24,
          7,
          6,
          30,
          66,
          5,
          43,
          43,
          23,
          64,
          6,
          18,
          7,
          37,
          38,
          28,
          20,
          19,
          9,
          25,
          120,
          88,
          22,
          76,
          21,
          126,
          22,
          34,
          10)
```

```
NOutcome = c(20,
             7,
             15,
             28,
             18,
             21,
             6,
             5,
             25,
             53,
             4,
             33,
             32,
             16,
             44,
             4,
             10,
             7,
             33,
             32,
             23,
             16,
             15,
             7,
             19,
             90,
             64,
             15,
             52,
             14,
             83,
             14,
             21,
             4)

Group = c(rep(1,17),rep(0,17))

dataKB = list(N = 34, y = NOutcome, x = Group, n = NTrials)

# Defining model for BRugs
model.lbmx = function(){
      for (i in 1:N) {
            y[i] ~ dbin(p[i],n[i])
            log(p[i]) <- (alpha0 + alpha1 * x[i] + u[i])
            u[i] ~ dnorm(0.0,tau)
      }
      # Priors
      alpha0 ~ dnorm(0, 1.0E-6)
      alpha1 ~ dnorm(0, 1.0E-6)
      sigma ~ dunif(0.01, 100)
      # Transforming standard deviation in precision
      tau<- 1/(sigma*sigma)
      # Transforming standard deviation in variance
      s2<- sigma*sigma

      RR <- exp(alpha1) # Calculating RR

      ICC <- s2/(s2+1) # Calculating ICC

      # Implementing constraint for probabilities between 0 and 1
```

```
      for (i in 1:N){
      ones[i] <- 1
      ones[i]  ~ dbern(C1[i])
      C1[i] <- step(1-p[i])
      }
}

# Defining initial values, it is always necessary to set initial
values for the random effects to ensure probabilities between 0 and 1
given the other initial values, it is indicated to put negative values

inits<- function(){
      list(alpha0 = -3, alpha1 = 0, sigma=1,u=rep(-3,34))
      list(alpha0 = -3, alpha1 = -1, sigma=1,u=rep(-3,34))
      list(alpha0 = -4, alpha1 = 1, sigma=1,u=rep(-3,34))
}

# Defining parameters
parameters<- c("alpha0", "alpha1", "RR", "s2","ICC")

# Running MCMC, the seed is to get identical results to the ones shown

library(BRugs)
simul<- BRugsFit(data=dataKB, inits=inits, para=parameters,
modelFile=model.lbmx, numChains=3, nIter=260000, nThin=1, nBurnin=0,
seed=9)

## Graphical Analysis

# Trajectory of the chains from the beginning
samplesHistory("*", mfrow=c(2,3))  # Produces Figure S1

# Trajectory of the chains after burn-in of 50000 iterations
samplesHistory("*", mfrow=c(2,3), beg=50001) # Produces Figure S2

# Gelman and Rubin for checking the burn-in chosen – exemplified only
for the intercept, needs coda package
library(coda)
a0=buildMCMC("alpha0")
gelman.plot(a0) # Produces Figure S3

a0wb=buildMCMC("alpha0",beg=50001)
gelman.diag(a0wb) # Obtaining GR statistics value

# Autocorrelation of the first chain without applying thin
samplesAutoC("*", chain=1, beg=50001) # Produces Figure S4

# Autocorrelation of the first chain after thin = 600 applied
samplesAutoC("*", chain=1, beg=50001, thin=600) # Produces Figure S5

# Getting estimates for the chosen burn-in period and thin
samplesStats("*",beg=50001,thin=600)

# Getting the mode only for intercept as an example, for the other
parameters, one just need to change the name of the parameter in the
buildMCMC function

a0 = buildMCMC("alpha0",beg=50001,thin=600)
sims = c(a0[[1]],a0[[2]],a0[[3]])
kernel0 = density(sims, bw="nrd0", n = 512, from=min(sims),
to=max(sims))
```

```
mode = kernel0$x[which.max(kernel0$y)]

# Getting plots of the posterior distributions
samplesDensity("*",mfrow=c(2,3),thin=600, beg=50001)
# Produces Figure S6
```

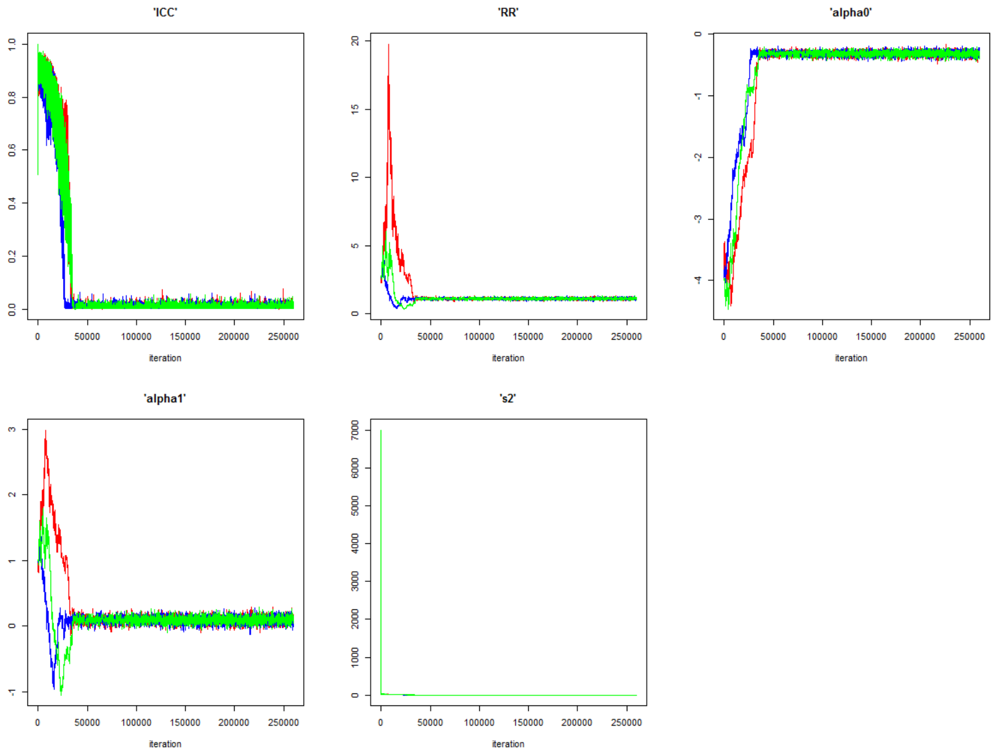

Figure S 1: Trajectory of the three chains for all iterations

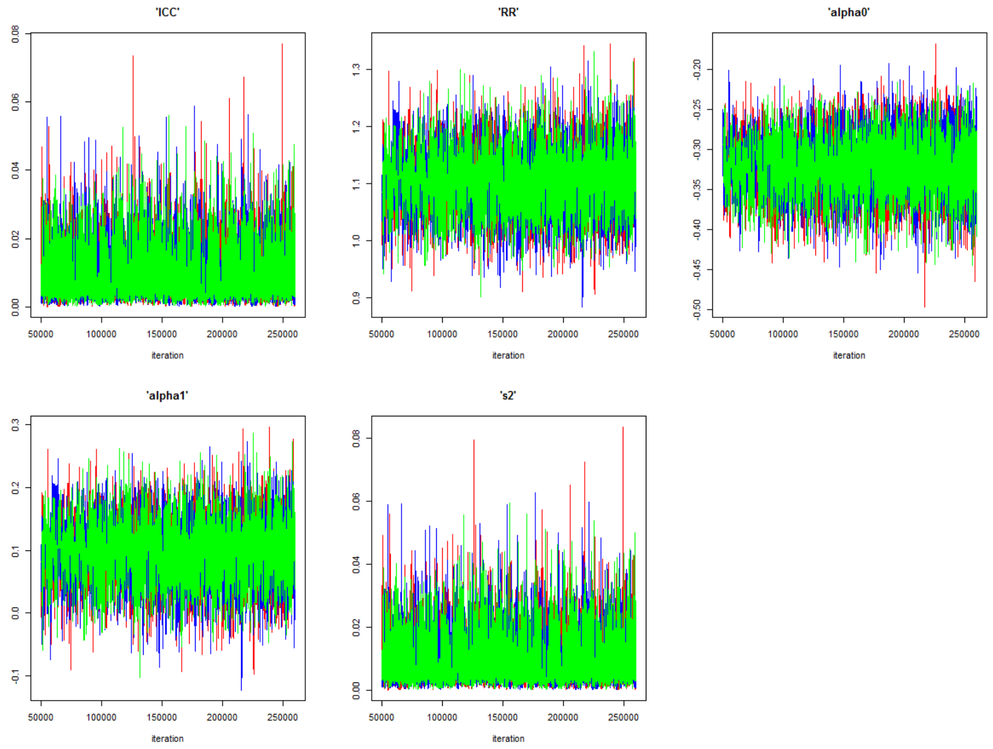

Figure S 2: Trajectory of the three chains after burn-in applied

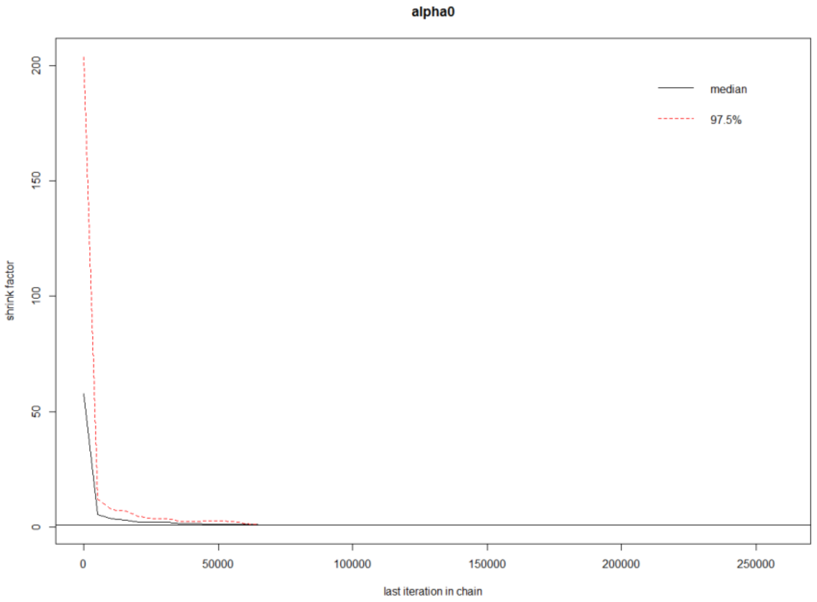

Figure S 3 Gelman and Rubin plot for all iterations

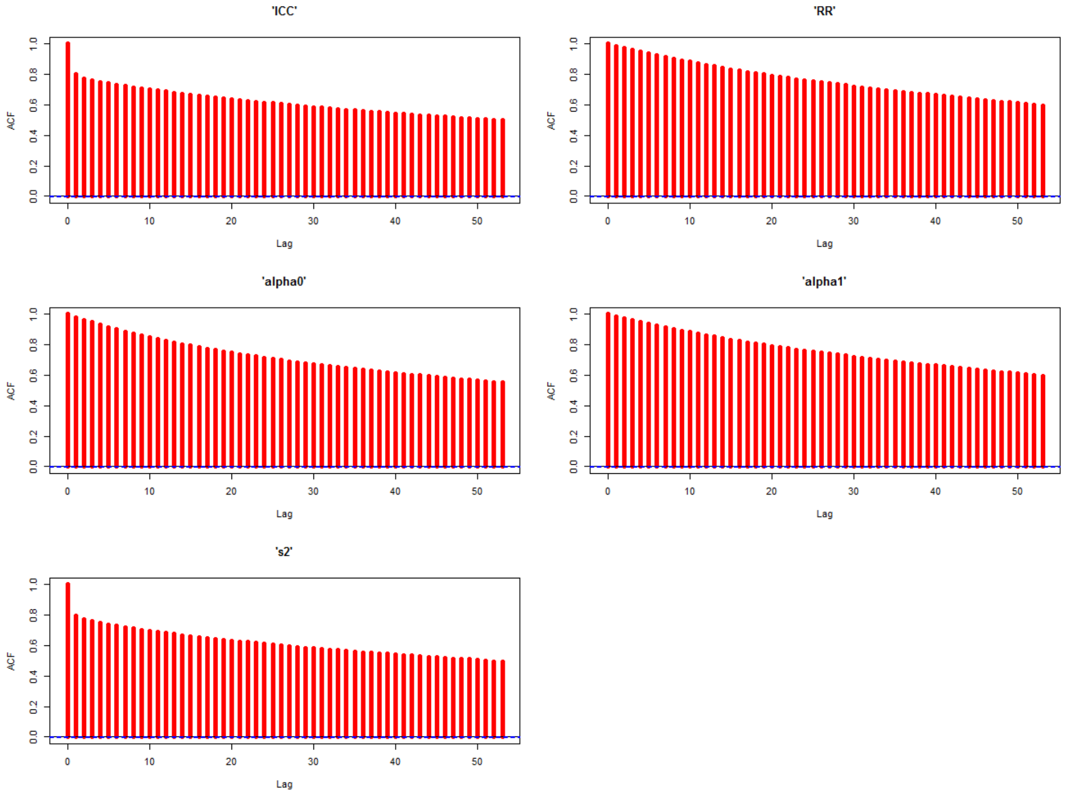

Figure S 4 Autocorrelation plot without thinning the chains

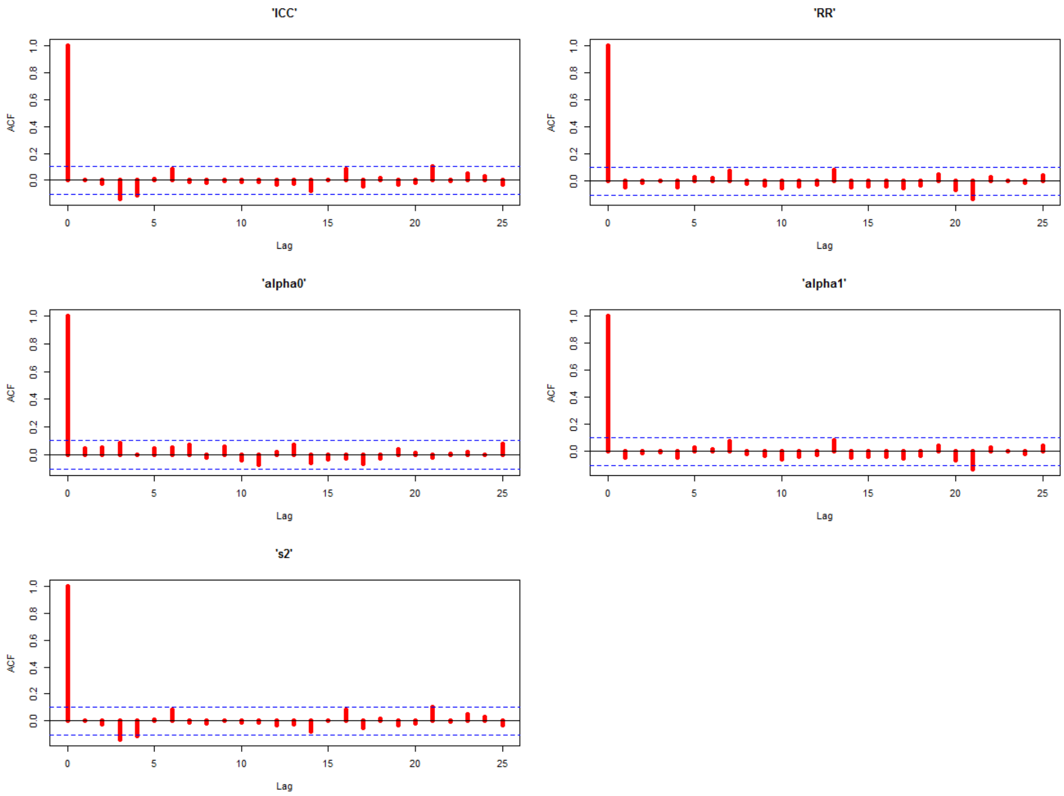

Figure S 5 Autocorrelation plot after thinning the chains with 600 iterations

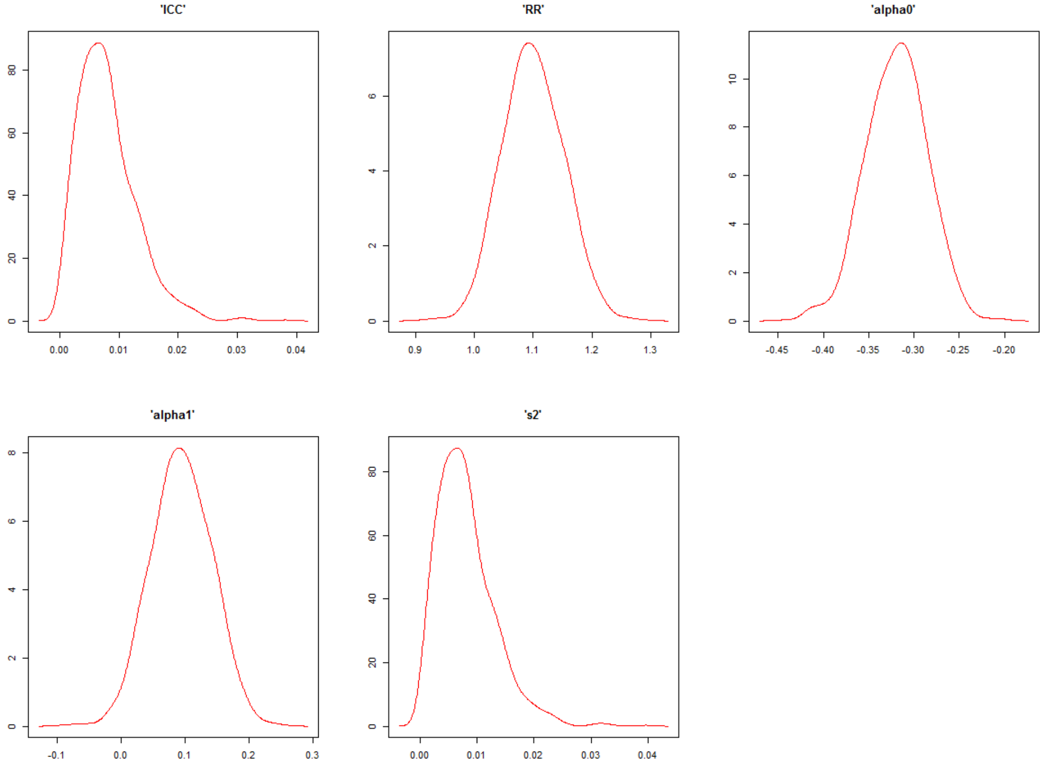

Figure S 6 Density of the posterior distributions

Comment on the Graphical Analysis: From Figure S1 it is possible to observe that the chains didn't have a stable behavior until the 50000th iteration for all parameters. Removing the 50000 first iterations, the produced chains had very similar behavior (Figure S2). Gelman and Rubin statistics seems to reach stability near the value 1 near the 50000th iteration, which agrees with the trajectory plots (Figure S3). Huge correlations are seen in Figure S4. After a sequence of trials, thinning of 600 iterations seemed successful in reducing the correlations after lag 1 (Figure S5). The posterior distributions for ICC and the variance of the random effects were the most skewed for this data (Figure S6), although it is not uncommon that the RR posterior be skewed too.

################### FREQUENTIST ANALYSIS IN SAS###################

* **Data Structure**: For those analyses an extended dataset was created, called blandext, where a binary outcome was created for each physician with the corresponding sucesses and failures.

```
* Random effects log-binomial - does not converge;
Proc glimmix data=blandext;
      class medico grupo;
      modeldesfecho = grupo /dist=bin link=log solution cl;
      randomint /sub=medico;
      covtest 'nulo' GLM /cl ;
run;

* GEE Poisson - exchangeable matrix;
Proc genmod data=blandext;
      class medico grupo;
      model desfecho = grupo /dist=poisson link=log cl ;
      estimate 'RR' gruporec 1 -1 /exp;
      repeated subject=medico/type=exch ;
run;

* GEE Log-binomial - exchangeable matrix;
Proc genmod data=blandext descending;
      class medico grupo;
      model desfecho = grupo /dist=bin link=log cl ;
      estimate 'RR' grupo 1 -1 /exp;
      repeated subject=medico/type=exch ;
run;
```

3 Code for data from the multilevel study on SUS satisfaction

```
###################### BAYESIAN ANALYSIS IN R #####################

# Model for BRugs
model.sus = function(){
      for (i in 1:12879) {
            y[i] ~ dbern(p[i])
            log(p[i]) <- (a0 + a1 * x1[i] + a2*x2[i]  + a3*x3[i] +
a4*x4[i] + a5*x5[i] + a6*x6[i]  + a7*x7[i] + a8*x8[i] + a9*x9[i] +
a10*x10[i]+ a11*x11[i] + a12*x12[i] + a13*x13[i] + a14*x14[i] +
a15*x15[i] + a16*x16[i] + a17*x17[i] + a18*x18[i] + a19*x19[i] +
a20*x20[i] + a21*x21[i] + a22*x22[i] + a23*x23[i] + a24*x24[i] +
u[municip[i]])
      }

      for(j in 1:61){
```

```
            u[j] ~ dnorm(0.0,tau)
      }

# Priors
a0 ~ dnorm(0, 1.0E-6)
a1 ~ dnorm(0, 1.0E-6)
a2 ~ dnorm(0, 1.0E-6)
a3 ~ dnorm(0, 1.0E-6)
a4 ~ dnorm(0, 1.0E-6)
a5 ~ dnorm(0, 1.0E-6)
a6 ~ dnorm(0, 1.0E-6)
a7 ~ dnorm(0, 1.0E-6)
a8 ~ dnorm(0, 1.0E-6)
a9 ~ dnorm(0, 1.0E-6)
a10 ~ dnorm(0, 1.0E-6)
a11 ~ dnorm(0, 1.0E-6)
a12 ~ dnorm(0, 1.0E-6)
a13 ~ dnorm(0, 1.0E-6)
a14 ~ dnorm(0, 1.0E-6)
a15 ~ dnorm(0, 1.0E-6)
a16 ~ dnorm(0, 1.0E-6)
a17 ~ dnorm(0, 1.0E-6)
a18 ~ dnorm(0, 1.0E-6)
a19 ~ dnorm(0, 1.0E-6)
a20 ~ dnorm(0, 1.0E-6)
a21 ~ dnorm(0, 1.0E-6)
a22 ~ dnorm(0, 1.0E-6)
a23 ~ dnorm(0, 1.0E-6)
a24 ~ dnorm(0, 1.0E-6)

# Variance transformations
sigma ~ dunif(0.01, 100)
tau<- 1/(sigma*sigma)
s2<- sigma*sigma


# Calculating PRs
RP1 <- exp(a1)
RP2 <- exp(a2)
RP3 <- exp(a3)
RP4 <- exp(a4)
RP5 <- exp(a5)
RP6 <- exp(a6)
RP7 <- exp(a7)
RP8 <- exp(a8)
RP9 <- exp(a9)
RP10 <- exp(a10)
RP11 <- exp(a11)
RP12 <- exp(a12)
RP13 <- exp(a13)
RP14 <- exp(a14)
RP15 <- exp(a15)
RP16 <- exp(a16)
RP17 <- exp(a17)
RP18 <- exp(a18)
RP19 <- exp(a19)
RP20 <- exp(a20)
RP21 <- exp(a21)
RP22 <- exp(a22)
RP23 <- exp(a23)
RP24 <- exp(a24)
```

```
# Implementing constraint
      for (i in 1:12879){
            ones[i] <- 1
            ones[i]  ~ dbern(C1[i])
            C1[i] <- step(1-p[i])
      }
}

# Centenring (ie, deducting the mean) continuous variables to improve convergence. Dichotomous variables (dummies) must be created previously for categorical variables.
data.sus = data.frame(municip=as.numeric(municipio),
x1=densidade1000-mean(densidade1000),
x2=perpopalfabetizada-mean(perpopalfabetizada),
x3=rendapc1000-mean(rendapc1000),
x4=pobreza-mean(pobreza),x5=idh-mean(idh),
x6=ubscemmilhab-mean(ubscemmilhab),x7=cobesf-mean(cobesf),
x8=idsus-mean(idsus),x9=idade1,x10=idade2,x11=idade3,x12=idade4,
x13=idade5,x14=cor01,x15=escol1,x16=escol2,x17=escol3,x18=escol4,
x19=atendidoondemora01,x20=fimatend1,x21=fimatend2,x22=tempo1,
x23=tempo2,x24=tempo3,y=comovoceavaliaoSUS)

# Defining initial values, it is always necessary to set initial values for the random effects to ensure probabilities between 0 and 1 given the other initial values, it is indicated to put negative values
inits<- list(
list(a0 = log(0.5),a1=0,a2=0,a3=0,a4=0,a5=0,a6=0,a7=0,a8=0,a9=0,
      a10=0,a11=0,a12=0,a13=0,a14=0,a15=0,a16=0,a17=0,a18=0,a19=0,
      a20=0,a21=0,a22=0,a23=0,a24=0,sigma=1,u=rep(-3,61)),
list(a0 = log(0.5),a1=0,a2=0,a3=0,a4=0,a5=0,a6=0,a7=0,a8=0,a9=0,
      a10=0,a11=0,a12=0,a13=0,a14=0,a15=0,a16=0,a17=0,a18=0,a19=0,
      a20=0,a21=0,a22=0,a23=0,a24=0,sigma=0.5,u=rep(-3,61)),
list(a0 = log(0.6),a1=0,a2=0,a3=0,a4=0,a5=0,a6=0,a7=0,a8=0,a9=0,
      a10=0,a11=0,a12=0,a13=0,a14=0,a15=0,a16=0,a17=0,a18=0,a19=0,
      a20=0,a21=0,a22=0,a23=0,a24=0,sigma=1,u=rep(-3,61))
)

# Defining parameters
params<- c("a0", "a1", "a2", "a3", "a4", "a5", "a6", "a7",
"a8", "a9", "a10", "a11", "a12", "a13", "a14", "a15", "a16", "a17",
"a18", "a19", "a20", "a21", "a22", "a23", "a24", "RR1", "RR2",
"RR3", "RR4", "RR5", "RR6", "RR7", "RR8", "RR9", "RR10",
"RR11", "RR12", "RR13", "RR14", "RR15", "RR16", "RR17", "RR18",
"RR19", "RR20", "RR21", "RR22", "RR23", "RR24", "s2")

# Running MCMC
simul<- BRugsFit(data=data.sus, inits=inits, para=params,
modelFile=model.sus, numChains=3, nIter=480000, nThin=400,
nBurnin=250000)

################### FREQUENTIST ANALYSIS IN SAS ###################

* Random effects logistic model;
Proc glimmix data=banco_final;
      classmunicipioidadeusuariocorusuarioescolaridadeusuarioatendidoondemorafimatendimentoquantotempoesperou;
      modelcomovoceavaliaoSUS(event='Regular/Ruim/MuitoRuim') =
densidade1000 perpopalfabetizada rendapc1000 pobreza
idhubscemmilhabcobesfidsusidadeusuariocorusuarioescolaridadeusuarioate
```

```
ndidoondemorafimatendimentoquantotempoesperou
/dist=binarysolutionoddsratio;
      randomint /sub=municipio;
      covtest 'null' GLM /cl ;
run;
```

4 Code for the low birth weight data

```
###################### BAYESIAN ANALYSIS IN R ######################

# Defining database for BRugs
# nobs = number of births
# nouts = number of categories of the polytomous outcome
# Y = matrix containing the dichotomous variables corresponding to
each outcome, the reference category must always be the last column
# X1 and X2 are the predictors, categorical predictors must always be
transformed in dicotomous variables (dummies)
# nx = number of predictors

datalogmul = list(nobs=189, nouts=4,
Y=cbind(Outcome_B,Outcome_C,Outcome_D,Outcome_A),X1=smoke,X2=age,
nx=2)

# Model for BRugs
Modellogmul <- function(){
      for (i in 1:nobs){
            Y[i, 1:nouts] ~ dmulti(p[i, 1:nouts], 1)
            for (j in 1:(nouts-1)) {
                  log(p[i,j]) <- b[j,1] + b[j,2]*X1[i] + b[j,3]*X2[i]
            }
            # Constraint for the probability of the reference category
            p[i,nouts] <- 1 - sum(p[i,1:(nouts-1)])
      }

      # Priors
      for(j in 1:(nouts-1)){
            for(k in 1:(nx+1)){
                  b[j,k] ~ dnorm(0, 1.0E-6)
            }
      }

      # Calculating RRs
      for(j in 1:(nouts-1)){
            for(k in 2:(nx+1)){
                  RR[j,k] <- exp(b[j,k])
            }
      }

      # Constraint for probabilities between 0 and 1
      for (i in 1:nobs){
            for(j in 1:(nouts-1)){
                  ones[i,j] <- 1
                  ones[i,j]  ~ dbern(C1[i,j])
                  C1[i,j] <- step(1-p[i,j])
            }
      }
}

# Defining parameters of interest
parameters<- c("b","RR")
```

```
# Setting initial values, which should ensure valid probabilities
inits = function(){
      list(b = matrix(c(-1.5,-1.5,-1.5,0,0,0,0,0,0),nrow=3))
      list(b = matrix(c(-2,-1.5,-1.5,0,0,0,0,0,0),nrow=3))
      list(b = matrix(c(-3,-1.5,-1.0,0,0,0,0,0,0),nrow=3))
}

# Running MCMC
simul<- BRugsFit(data=datalogmul,  para=parameters, inits=inits,
modelFile=modellogmul, numChains=3, nIter=3012000, nThin=3000,
nBurnin=30000)

#################### FREQUENTIST ANALYSIS IN R ####################

# Same code used for the ADHF patients data, with each of the binary
outcomes created
```

5 ICC demonstration for random effects log-binomial model

Be the random effects log-binomial model with only one predictor, for simplicity and without loss of generality:

$$\theta_{ij} = P(Y_{ij} = 1|X_{ij}, u_i) = \exp\left(\beta_0 + \beta_1 X_{ij} + u_i\right)$$

where Y_{ij} is the binary outcome for the *j*-th unit in the *i*-th cluster, with Bernoulli distribution, and X_{ij} is the value of the predictor for this observation and u_i is the random effect of the *i*-th *cluster*, for which it is assumed that $u_i \sim N(0, \sigma_u^2)$. Suppose the binary outcome originally came from a categorization of a continuous latent variable Y_{ij}^*, such that $Y_{ij} = 1$ if $Y_{ij}^* \geq 0$ and $Y_{ij} = 0$ otherwise. The latent variable model equivalent to the previous equation is given by:

$$Y_{ij}^* = \beta_0 + \beta_1 X_{ij} + u_i + \varepsilon_{ij}$$

where $\varepsilon_{ij} \sim Exponential(1)$. This is because,

$$\theta_{ij} = P\left(Y_{ij} = 1 \middle| X_{ij}, u_i\right) = P\left(Y_{ij}^* \geq 0 \middle| X_{ij}, u_i\right)$$

$$= P\left(\varepsilon_{ij} \geq -(\beta_0 + \beta_1 X_{ij} + u_i) \middle| X_{ij}, u_i\right) = \exp(\beta_0 + \beta_1 X_{ij} + u_i).$$

Thus, the correlation between two units within the same cluster for the latent variable is given by:

$$ICC_{log-bin} = \frac{\sigma_u^2}{\sigma_u^2 + 1}$$

since the variance of ε_{ij} also equals 1.
